# Thermoresponsive Polyphosphoester via Polycondensation Reactions: Synthesis, Characterization, and Self-Assembly

**DOI:** 10.3390/molecules27186006

**Published:** 2022-09-15

**Authors:** Yoshihiro Yamakita, Issei Takeuchi, Kimiko Makino, Hiroshi Terada, Akihiko Kikuchi, Kolio Troev

**Affiliations:** 1 Faculty of Pharmaceutical Science, Tokyo University of Science, 2641 Yamazaki, Noda, Chiba 278-8510, Japan; 2 Faculty of Pharmaceutical Science, Josai International University, 1 Gumyo, Togane, Chiba 283-8555, Japan; 3 Department of Materials Science and Technology, Faculty of Advanced Engineering, Tokyo University of Science, 6-3-1 Niijuku, Katsushika, Tokyo 125-8585, Japan; 4 Institute of Polymers, Bulgarian Academy of Sciences, 1113 Sofia, Bulgaria

**Keywords:** polyphosphoesters, amphiphilic polymers, thermoresponsive polymers, micelles, drug delivery

## Abstract

Using a novel strategy, amphiphilic polyphosphoesters based on poly(oxyethylene H-phosphonate)s (POEHP) with different poly(ethylene glycol) segment lengths and aliphatic alcohols with various alkyl chain lengths were synthesized using polycondensation reactions. They were characterized by ^1^H NMR, ^13^C {H} NMR ^31^P NMR, IR, and size exclusion chromatography (SEC). The effects of the polymer structure on micelle formation and stability, micelle size, and critical micelle temperature were studied via dynamic light scattering (DLS). The hydrophilic/hydrophobic balance of these polymers can be controlled by changing the chain lengths of hydrophilic PEG and hydrophobic alcohols. A solubilizing test, using Sudan III, revealed that hydrophobic substances can be incorporated inside the hydrophobic core of polymer associates. Loading capacity depends on the length of alkyl side chains. The results obtained indicate that these structurally flexible polymers have the potential as drug carriers.

## 1. Introduction

Most anticancer drugs used today have serious side effects due to their nonselective distribution in the body. The problem is a nonselective attack on normal cells, which causes various side effects, such as canker sores, hair loss, vomiting, anemia, and leucopenia. A promising approach to improve some characteristics of low molecular weight drugs, already approved and used in practice, as well as to impart new valuable properties is the macromolecular approach, i.e., application of appropriate polymers for drug immobilization, chemically conjugated or physically bound to a polymer chain. Polyphosphoesters, a family of biodegradable substances through hydrolysis and possibly through enzymatic digestion of phosphate linkages under physiological conditions and biocompatible polymers including poly(alkylene H-phosphonate)s and their derived polyphosphates and polyphosphoramidates, are promising polymers for drug delivery. Recently, polyphosphoesters were identified to induce a similar stealth effect as PEG [1]. Polymeric micelles, over the past decades, have drawn considerable interest because of their great potential in anticancer drug delivery and diagnostic imaging applications [2,3,4,5]. A variety of drugs have been encapsulated into the hydrophobic core of polymeric micelles to treat various diseases [6,7,8,9]. It was shown that the hydrophobicity of the micellar core affects the antitumor efficacy of hydrophobic polyphosphoester-based micelles, which possess different alkyl side chain lengths. After the encapsulation of hydrophobic doxorubicin, the drug release rate was also related to the hydrophobicity of the micellar core. Although DOX-loaded micelles with the lowest hydrophobic core showed the most powerful inhibition in vitro, the increased hydrophobicity of micellar core significantly enhanced tumor growth inhibition in an MDA-MB-231 tumor model due to the prolonged circulation time, enhanced drug retention in tumors, and reduced unnecessary drug loss in tumors microenvironment [10].

Amphiphilic *co*-, graft polyphosphoesters [11,12,13], and polymers based on polyphosphoesters and: poly(ethylene glycol) [14,15]; poly(L-lactic acid) [16,17]; poly(ε-caprolactone) [18,19,20,21,22,23,24,25,26,27,28]; poly(hydroxybutyrate [29]; folic acid-paclitaxel [30]; poly(ethylene oxide)-paclitaxel [31,32]; poly(ethylene glycol)-doxorubicin [33]; and poly [2-(dimethylamino)ethyl methacrylate] [34] were described and used as carriers of hydrophobic drugs. There is no data in the literature on amphiphilic polyphosphoesters prepared via the polycondensation process. Polycondensation as a process for the preparation of amphiphilic polyphosphoesters has big advantages compared to the polymerization process, namely, because (i) different starting hydroxyl-containing compounds can be used; (ii) synthesis can proceed without a catalyst; (iii) there is no need for the purification of the reaction product; (iv) degraded products can be designed in advanced; (v) copolymers can be obtained; and (vi) there are commercially available starting monomers.

Poly(oxyethylene H-phosphonate)s are multifunctional drug carriers, capable of converting into: (i) hydrophilic; (ii) hydrophobic; (iii) amphiphilic, and (iv) stimuli-responsive poly(alkyloxyethylene phosphate)s. The drug can be carried: (v) chemically (covalent bond), due to the presence of a highly reactive P-H group [35,36,37,38]; (vi) via ionic bonds (P-OH group) [37,39]; (vii) physically, due to the presence of a strong proton acceptor—P=O group [36,37,39]; (viii) or via micelles.

The effect of hydrophilic and hydrophobic chain lengths of poly(alkyloxyethylene phosphate)s on micelle formation, micelle size, critical micelle temperature, micelle stability at body temperature, and micelle average diameter depending on solution, temperature, and polymer concentration are studied.

## 2. Results and Discussion

### 2.1. Synthesis of Poly(oxyethylene H-phosphonate)s

**Scheme 1:**
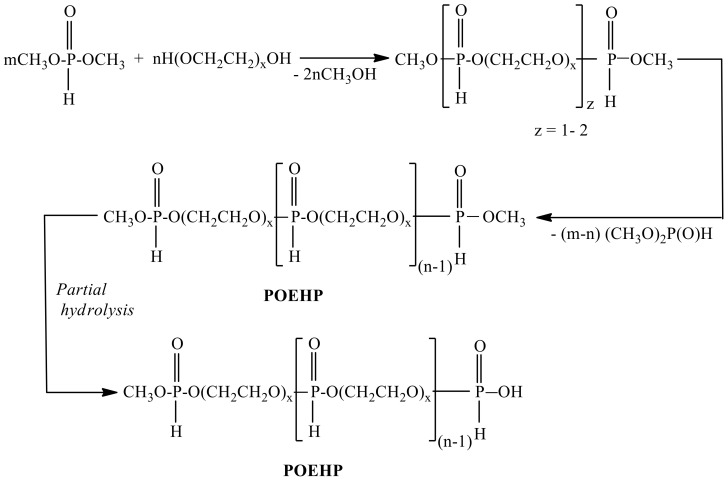
Synthesis of poly(oxyethylene H-phosphonate) (POEHP).

Poly(oxyethylene H-phosphonate)s were synthesized via a two-step polycondensation reaction of dimethyl H-phosphonate and poly(ethylene glycol)s, with nominal molecular weights of 200, 300, 400, and 600 Da. At the first stage, oligooxyethylene H-phosphonate with phosphonate end groups (z = 1–2) was obtained. At the second stage at 160 °C, there proceeds a disproportionation of oligooxyethylene H-phosphonate (it can be considered as a nonsymmetrical diester of H-phosphonic acid) to furnish poly(oxyethylene H-phosphonate) (Scheme 1).

Polymers were characterized by ^1^H NMR, ^13^C{H}NMR, and ^31^P NMR spectroscopy. In the ^1^H NMR spectrum (Figure S1) signal at δ ppm: 6.95, d, with ^1^J(P,H) = 715.98 Hz can be assigned to the P-H proton in the following unit -CH_2_OP(O)(*H*)OCH_2_-); 6.87, d, with ^1^J(P,H) = 708.66 Hz can be assigned to the P-H proton in the following segment CH_3_OP(O)(*H*)OCH_2_-); 6.84, d, with ^1^J(P,H) = 694.76 Hz can be assigned to the P-H proton in the following segment HOP(O)(*H*)OCH_2_-); multiplet in the area 4.13–4.30 is characteristic for-OP(O)(H)OC*H*_2_) protons and those at 3.38–3.83 is characteristic for -OC*H*_2_C*H*_2_O-protons. In ^13^C{H} NMR spectrum (Figure S2) signals at: 64.57 (d, ^2^J(P,C) = 6.00 Hz, 70.03 (d, ^3^J(P,C) = 5.79 Hz, and 70.43 (s) can be assigned to the carbon atoms in the following segments -*C*H_2_OP(O)(H)O*C*H_2_-:), -OP(O)(H)OCH_2_*C*H_2_-), and-O*C*H_2_*C*H_2_O-), respectively. In ^31^P{H} NMR spectrum (Figure S3) there are signals for three types of phosphorus atoms at ppm: 11.17 ppm; 10.46 ppm; and 8.36 ppm.

^31^P NMR spectrum (Figure S4) revealed that signals at δ: 11.17 represents doublet of sextets with ^1^J(P,H) = 708.7 Hz and ^3^J(P,H) = 11.9 Hz; Additionally, 10.46, doublet of quintets with ^1^J(P,H) = 715.9 Hz and ^3^J(P,H) = 9.8 Hz and at 8.36, dt with ^1^J(P,H) = 697.02 Hz, ^3^J(P,H) = 10.96 Hz, and have to be assigned to the phosphorus atoms with the following substituents -CH_2_O*P*(O)(H)OCH_3_, -CH_2_O*P*(O)(H)OCH_2_-, and -CH_2_O*P*(O)(H)(OH), respectively. Molecular weight (M*_n_*, M*_w_*), polydispersity index (PDI), and degree of polymerization (DP) were determined by SEC and summarized in Table 1. The results showed that DP varied from 14.8 to 25.8 and PDI varied from 1.32 to 1.83 depending on the type of PEG.

According to the novel strategy, the first step of the preparation of amphiphilic polyphosphoesters is the synthesis of poly(oxyethylene H-phosphonate)s. From this point of view, the advantages of the polycondensation process make the novel strategy attractive, with big potential, without any limitations concerning the design of the structure and composition of the amphiphilic polyphosphoesters compared to the polymerization process. The big disadvantage of polymerization for the preparation of polyphosphoesters-poly(alkylene H-phosphonate)s and poly(alkylalkylene phosphate)s arises from the limitation of the starting monomers. There are only two starting monomers-5-, and 6-members cyclic esters of H-phosphonic and phosphoric acids and as result, it is possible to obtain poly(ethylene (or propylene) H-phosphonate)s and poly(alkylethylene (or propylene) phosphate)s. There are no limitations when poly(alkylene H-phosphonate)s are used as a precursor for the synthesis of the corresponding poly(alkylalkylene phosphate)s. Poy(alkylalkylene phosphates) with practically quantitative yield in mild conditions are prepared due to the high reactivity of the P-H group of poly(alkylene H-phosphonate)s toward hydroxyl and amino groups [40].

### 2.2. Synthesis of Poly(alkyloxyethylene phosphate)s (POEAP)

**Scheme 2:**
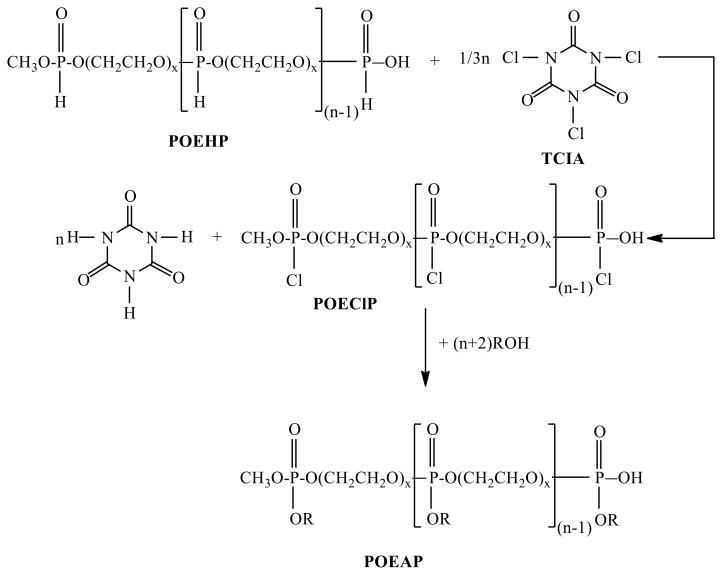
Synthesis of poly(alkyloxyethylene phosphate) (POEAP).

Poly(alkyloxyethylene phosphate) was synthesized in a two-step reaction from poly(oxyethylene H-phosphonate), trichloroisocyanuric acid, and alcohol [41] (Scheme 2).

In the first step, poly(oxyethylene H-phosphonate) was converted to the corresponding poly(oxyethylene chlorophosphate) using trichloroisocyanuric acid as a chlorination agent [39]. In the second stage, the poly(oxyethylene chlorophosphate) was added to aliphatic alcohol (Table 2). The completion of the oxidation and substitution reactions was controlled by the ^31^P{H} NMR spectroscopy. The chemical shift of the phosphorus atom, bonded with the hydrogen atom in the repeating unit is at 10.45 ppm whereas the phosphorus atom bonded to chlorine is at 5.68 ppm (Figure S7). The degree of oxidation based on the ^31^P{H}NMR spectroscopy is 100%. In ^1^H NMR spectrum the multiplet 4.14–4.10 ppm is characteristic for C*H*_2_OP(O)(OCH_3_)OC*H*_2_) protons; doublet at 3.71 with ^3^J(P,H) = 11.14 Hz have to be assigned to POC*H*_3_ protons; multiplet at 3.66–3.58 is for C*H*_2_OC*H*_2_-protons. The presence of the doublet at 3.71 ppm confirms the proceeding of the alkylation reaction. In ^13^C{H}NMR spectrum doublet at 55.36 ppm is characteristic for a carbon atom in PO*C*H_3_ group. Signals at 70.03 (d, ^3^J(P,C) = 5.8 Hz and at 64.57 (d, ^2^J(P,C) = 6.2 Hz have to be assigned to carbon atoms in the following segments -POCH_2_*C*H_2_- and -PO*C*H_2_CH_2_-, respectively. The signals in ^31^P{H} NMR spectrum (Figure S5) at: 2.37 ppm (1.97%); 1.28 ppm (96.4%); 0.57 ppm (1.63%) are characteristic for phosphorus atoms in phosphate structure. The signal at 1.28 ppm in ^31^P NMR spectrum (Figure S6) represents octet with ^3^J(P,H) = 10.38 and 6.71 Hz and has to be assigned to phosphorus atom in the repeating unit -CH_2_O*P*(OCH_3_)(O)OCH_2_-. The ^3^J(P,H) constant with a value of 10.38 Hz is characteristic for P-OC*H*_3_ protons while those with a value of 6.71 Hz are characteristic for C*H*_2_O*P*(OCH_3_)(O)OC*H*_2_-protons. The degree of substitution (alkylation) reaction is based on the ratio between integral intensities of the signals at 1.28, 2.37, and 0.57 ppm (Figure S5) (the chemical shift for phosphorus atoms with phosphate structures) and at 5.66 ppm. In ^31^P{H}NMR spectrum there is no signal at 5.66 ppm, but this does not mean that the degree of alkylation is 100%, since some of the P-Cl groups were converted to the P-OH group by hydrolysis (chemical shift for this phosphorus atom is at 0.57 ppm). In this paper, polymer type was symbolized. When the code was P*_a_*-*b*, *a* means the molecular weight of PEG used for the preparation of POEHP, and *b* means the type of side chains.

The relationship between hydrophilic/hydrophobic balance and micelle diameter was investigated with DLS. The diameter of poly(alkyloxyethylene phosphate)s micelles depends on the length type of the PEGs (Table 3). Increasing the PEGs length leads to a strong decrease in the diameter of the micelles. This result is expected because PEG 600 is more water-soluble compared to others. Micelle diameter depends on the alkyl chain length (Table 4). For polymers in which the carbon chain length of the pendant group is smaller than that of the octyl group, the micellar diameter could not be measured. We considered that they were dissolved in water. The results revealed that poly(alkyloxyethylene phosphate)s with carbon chain length in the pendant group longer than or equal to the octyl group self-assembled into the a micellar structure in an aqueous solution. When the alkyl chain lengthened from octyl to dodecyl, micelles became smaller and more stable because hydrophobic interaction became stronger with the increasing alkyl chain length. By contrast, as PEG chains lengthened, coagulation power became smaller. When PEG chains changed from 400 Da to 600 Da, the polymers became more water-soluble and harder to make micelles at room temperature.

### 2.3. Micelle Stability

#### 2.3.1. Dependence of Micelle Diameter on the Alkyl Chain Length and the Polymer Concentration

**Figure 1:**
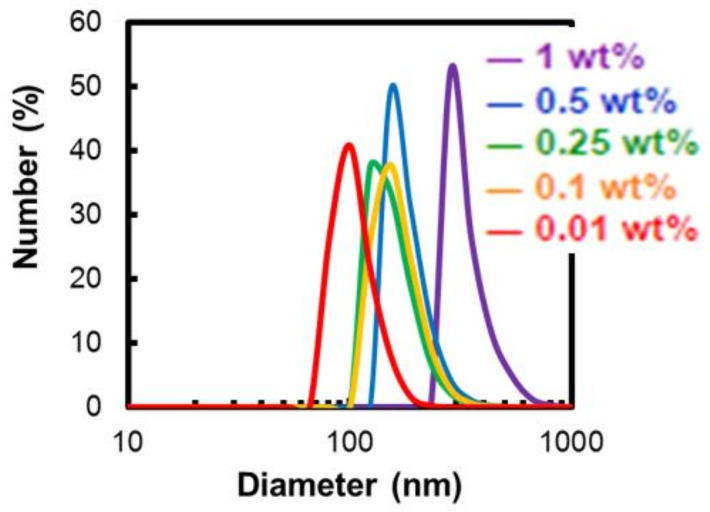
Effects of P_400_-OC_8_H_17_ concentration on micelle size distribution.

**Figure 2:**
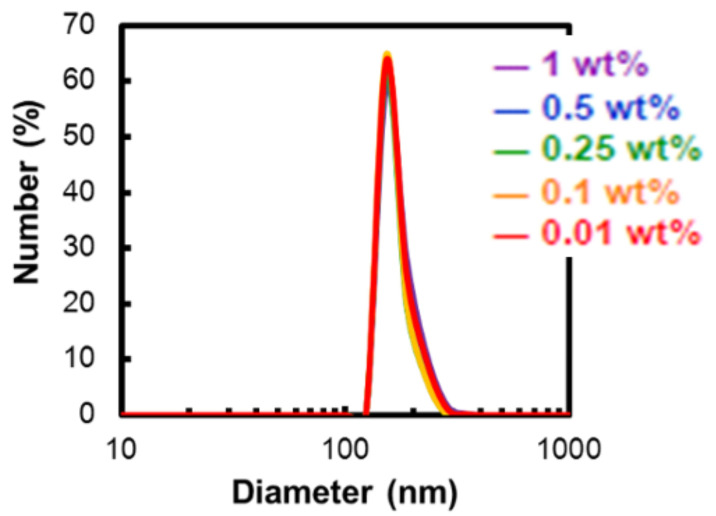
Effects of P_400_-OC_10_H_21_ concentration on micelle size distribution.

**Figure 3:**
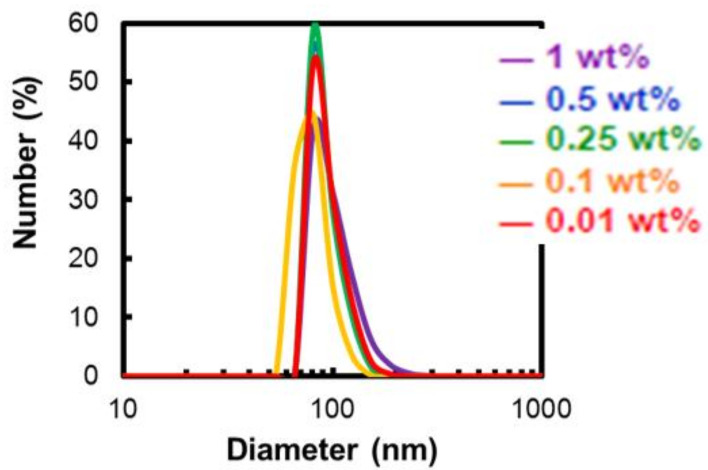
Effects of P_400_-OC_12_H_25_ concentration on micelle size distribution.

The DLS study of the dependence of the micelle diameter on the alky chain length and polymer concentration revealed that the micelle size of poly(octyloxyethylene phosphate) (Table 5 and Figure 1) became larger with increasing concentration, while in the case of poly(decyloxyethylene phosphate), micelles were stable even at 1 wt% (Table 5 and Figure 2). The size of the micelles using poly(dodecyloxyethylene phosphate) was also stable regardless of the concentration. However, there was some variation compared to poly(decyloxyethylene phosphate) micelles (Figure 3). In addition, it can be large at concentrations above 1 wt%.

#### 2.3.2. Temperature Dependence of Micelle Diameter from the Temperature and PEG’s Chain Length

DLS study of the effect of temperature on micelle size (Table 6) revealed that poly(dodecyloxyethylene phosphate)s, based on PEG 200 and PEG 300 formed micelles even at temperatures higher than 25 °C because heating the polymer solution resulted in reduced water solubility of PEG chains when dehydrated. The DLS study revealed that temperature has little effect on micelle size. Poly(dodecyloxyethylene phosphate)s, based on PEG 400 represent stimuli-responsive polymer. At 25 °C the polymer did not create micelles, but increasing the temperature to at least 30–40 °C started self-assembly. Decreasing the temperature caused the polymer to become soluble. Polymeric micelles based on PEG 200 and PEG 300 are expected to be stable after the intracorporal injection.

#### 2.3.3. Solubilizing Test

**Figure 4:**
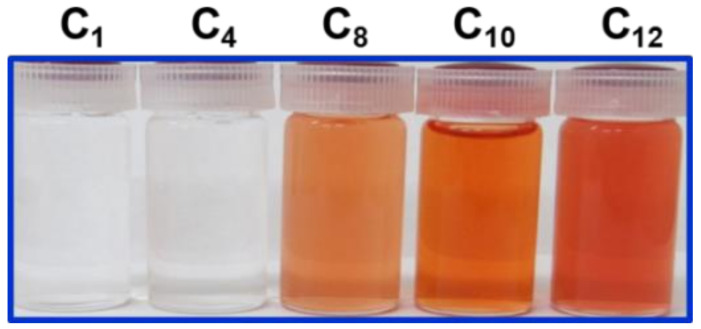
Differences in solution appearance depend on the carbon chain length of the polymer when dissolving Sudan III.

**Figure 5:**
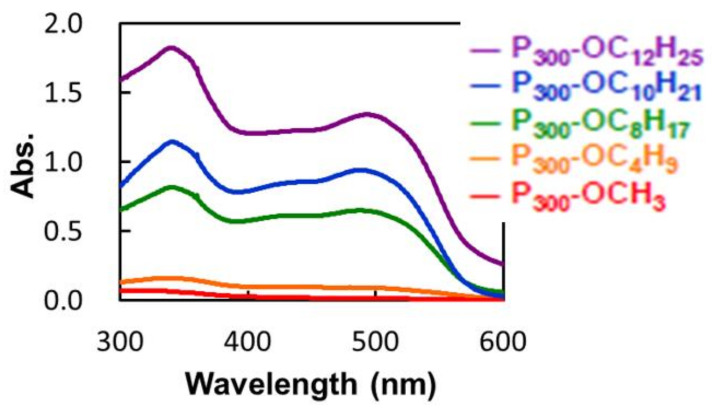
Absorption spectra at wavelengths of 300–600 nm when Sudan III was dissolved in polymer solutions with different carbon chain lengths.

Sudan III is a hydrophobic pigment and has an absorption maximum of 507 nm. Therefore, it does not dissolve in water and can be eliminated from the solution by filtration. The appearance of the prepared solution is shown in Figure 4, and the absorption spectrum from 300 to 600 nm is shown in Figure 5, and absorbance values at 500 nm were summarized in Table 7.

The poly(alkyoxyethylene phosphate)s have no absorption at 500 nm themselves. The results of the control experiment indicated that Sudan III had little solubility in water. The absorbance of the prepared polymer solutions was greater than the control solution. This revealed that Sudan III was incorporated inside the hydrophobic core of the polymer associates. More pigment was incorporated into the micelle with the increase in the length of the alkyl chain, e.g., the hydrophobicity of the poly(alkyloxyethylene phosphate)s affected the solubilizing capacity. Since the main purpose was to confirm whether the dye was solubilized by the polymer, data was obtained by a simple method. Although the values of solubilizing capacity shown in Table 7 are small, they may be improved by dissolving Sudan III in an organic solvent rather than in powder form.

## 3. Experimental Section

### 3.1. Materials

Dimethyl H-phosphonate (DMP; 98%) from Tokyo Chemicals Industries (Tokyo, Japan) was purified via vacuum distillation at 60 °C under vacuum (20 mmHg) and stored under nitrogen atmosphere in round-bottomed flasks equipped with pressure/vacuum glass stopcocks. Poly(ethylene glycol) (PEG) from Wako Pure Chemicals (Osaka, Japan), with nominal molecular weights of 200, 300, 400, and 600 Da was dried just before use by refluxing with toluene with Dean-Stark trap for 0.5 h followed by azeotropic distillation at 60 °C under vacuum (50 mmHg), and then 80 °C under dynamic vacuum. Acetonitrile from Wako Pure Chemicals was purified via distillation and stored under a nitrogen atmosphere in round-bottomed flasks equipped with pressure/vacuum glass stopcocks. *N*, *N*-dimethylformamide (DMF; ACS/HPLC certified, <0.001% H_2_O) was obtained from Wako Pure Chemicals and used as received. Chloroform-d (99.8%, containing 0.05 vol% TMS) was purchased from Wako Pure Chemicals and stored refrigerated before use. Toluene, methanol, 1-Butanol, 1-Octanol, 1-Decanol, 1-Dodecanol, hexane, chloroform, and lithium chloride (LiCl) were obtained from Wako Pure Chemicals and used as received. Trichloroisocyanuric acid (TCIA) was obtained from Sigma-Aldrich (St. Louis, MO, USA) and used as received. Sudan III was obtained from Tokyo Chemicals Industries and used as received. Water used in this study was purified with Milli-Q A10 (Millipore, Billerica, MA, USA) unless otherwise mentioned.

### 3.2. Instrumentation

All ^1^H NMR and ^31^P NMR spectra were recorded on a Bruker AVANCE NMR Spectrometer, (JEOL, Tokyo, Japan) operating at 250 MHz at room temperature in CDCl_3_ or CD_3_OD with TMS and with phosphoric acid as the internal standard, respectively.

The size distribution of micelles was determined by the dynamic light scattering (DLS) method using an ELS-Z, 2PDDS (OTSUKA ELECTRONICS, Hirakata, Japan).

UV-visible spectra were obtained using a multipurpose UV-Vis Spectrophotometer, MPS-2450 (SHIMAZU, Kyoto, Japan).

The molecular weights (MW) and polydispersity index (PDI) of polymers were determined by size exclusion chromatography (SEC) (TSK-GEL G5000HHR 7.8mm I.D.X 300 mm column) (JASCO, Tokyo, Japan) using PEG standard and employing an RI detector. DMF with LiCl, 10 mL at 40 °C was used as eluent (flow rate: 1.0 mL/min).

### 3.3. Synthesis of Poly(oxyethylene H-phosphonate)s

Poly(oxyethylene H-phosphonate)s (POEHP) were obtained from commercial dimethyl H-phosphonate (DMP) and poly(ethylene glycol)s (PEG) with nominal molecular weights (200, 300, 400, and 600 Da) in a two-stage polycondensation process [42,43,44,45]. The entire synthesis was carried out under an inert atmosphere. A typical synthetic procedure is described as follows: Purified DMP (2.51 g, 2.28.10^−2^ mol) and PEG 400 (7.1 g, 1.78.10^−2^ mol) were added to a two-necked round-bottomed flask, equipped with a magnetic stirring bar, inlet adaptor for inert gas, and vacuum condenser. In the first stage, dry nitrogen was passed through the reaction mixture, and the temperature was increased to 135 °C. Methanol evolution began a few minutes after the reaction temperature reached 135 °C. The reaction was kept at this temperature for 5 h. Dynamic vacuum (1 mmHg) was applied with the temperature increasing to 160 °C. Notable bubble generation was observed in the reaction mixture followed by distillation of a colorless liquid (methanol and DMP). After 4 h, the reaction product was stopped from heating but kept stirring for more than 30 min with a dynamic vacuum. Then, the vacuum was released under dry nitrogen flow. Using the same procedure, poly(oxyethylene H-phosphonate)s based on PEG 200, 300, 400, and 600 Da were synthesized. The products obtained from the polycondensation of DMP with PEG 200, 300, 400, and 600 Da were viscous liquids. The reaction products were characterized by ^1^H, ^13^C{H}NMR, and ^31^P NMR spectroscopy. Molecular weights and polydispersity index (PDI) of all POEHP were measured independently by SEC in DMF. Here, we report NMR data for POEHP based on PEG 400: NMR data for POEHP based on PEG 200, 300, and 600 are similar.

^1^H NMR (CDCl_3_) (Figure S1) δ ppm: 6.95 (d, ^1^J(P,H) = 715.98 Hz, -CH_2_OP(O)(H)OCH_2_-); 6.87 (d, ^1^J(P,H) = 708.66 Hz, CH_3_OP(O)(H)O-); 6.84 (d, ^1^J(P,H) = 694.76 Hz, HOP(O)(H)OCH_2_-); 4.13–4.30 (m, -OP(O)(H)OCH_2_) and 3.38–3.83 (m, -OCH_2_CH_2_O-).

^13^C{H} NMR (CDCl_3_) (Figure S2) δ ppm: 64.57 (d, ^2^J(P,C) = 6.00 Hz, -CH_2_OP(O)(H)OCH_2_-:); 70.03 (d, ^3^J(P,C) = 5.79 Hz, -OP(O)(H)OCH_2_CH_2_-) and 70.43 (s, -OCH_2_CH_2_O-).

^31^P{H} NMR (CDCl_3_) (Figure S3) δ ppm: 11.17 ppm; 10.46 ppm; 8.36 ppm.

^31^P NMR (CDCl_3_) (Figure S4) δ ppm: 11.17 (d sextet, ^1^J(P,H) = 708.7 Hz, ^3^J(P,H) = 11.9 Hz, -CH_2_OP(O)(H)OCH_3_); 10.46 (d quintet; ^1^J(P,H) = 715.9 Hz and ^3^J(P,H) = 9.8 Hz, -CH_2_OP(O)(H)OCH_2_-); and 8.36 (dt; ^1^J(P,H) = 697.02 Hz, ^3^J(P,H) = 10.96 Hz, -CH_2_OP(O)(H)OH).

### 3.4. Synthesis of Poly(alkyloxyethylene phosphate)s

Poly(alkyloxyethylene phosphate)s were obtained from poly(oxyethylene H-phosphonate), trichloroisocyanuric acid (TCIA), and alcohol (methanol, n-butanol, octanol, decanol, and dodecanol) in a one-pot synthesis. The entire synthesis was carried out under an inert atmosphere. A typical synthetic procedure is described as follows: POEHP-based on PEG 400 (2.26 g, 0.005 mol) was added to a two-necked round-bottomed flask, equipped with a magnetic stirring bar and inlet adaptor for inert gas, and dissolved by 5 mL of acetonitrile. To this solution, TCIA (0.39 g, 0.0017 mol) was added with stirring. The reaction mixture was kept for 5h at 35 °C and methanol (0.16 g, 0.005 mol) was added to the solution under the same condition. A typical purification procedure is described as follows: First, the obtained solution was filtered with a 0.45 μm syringe-driven filter. Then, the solvent was evaporated by distillation. After that, the product was dissolved in 10 mL of chloroform and stirred for several hours. The solution was filtered with a 0.45 μm syringe-driven filter and then, reprecipitated by hexane twice. Finally, the resultant poly(alkyloxyethylene phosphate) was dried thoroughly at 40 °C in vacuo and characterized by ^1^H, ^13^C{H}, ^31^P{H}NMR spectroscopy, and IR spectroscopy.

Spectroscopic data for poly(methyloxyethylene phosphate) based on PEG 400:

^1^H NMR (CD_3_OD) δ (ppm): 4.14–4.10 (m, CH_2_OP(O)(OCH_3_)OCH_2_), 3.71 (d, ^3^J(P,H) = 11.14 Hz, POCH_3_), 3.66−3.58 (m, CH_2_OCH_2_).

^13^C{H} (CD_3_OD), δ (ppm): 70.43 (CH_2_OCH_2_), 70.03 (d, ^3^J(P,C) = 5.8 Hz, POCH_2_CH_2_), 64.57 (d, ^2^J(P,C) = 6.2 Hz, POCH_2_CH_2_), 55.36 (d, ^2^J(P,C) = 5.9 Hz,POCH_3_.

^31^P{H} NMR (CD_3_OD) (Figure S5) δ (ppm): 2.37 (2.1%); 1.28 (88.1%); 0.57 (9.8%).

^31^P NMR (CD_3_OD) (Figure S6) δ (ppm): 2.37, m, CH_3_OP(O)(OCH_3_)OCH_2_-; 1.28, octet, ^3^J(P,H) = 10.38 and 6.71 Hz, -CH_2_OP(OCH_3_)(O)OCH_2_-; 0.57, m, -H_2_CO-P(O)OCH_3_(OH)

IR (KBr, cm^−^^1^): 1258 (P=O) (stretching); 1103 (P-OCH_2_- and P-OCH_3_), (CH_2_OCH_2_) (stretching).

Spectroscopic data for poly(butyloxyethylene phosphate) based on PEG 400:

^1^H NMR (CD_3_OD), δ (ppm): 0.91, CH_3_CH_2_-(t, ^3^J(H,H) = 5.7 Hz); 1.31–1.36, CH_3_CH_2_-CH_2_-(m); 1.49–1.53, CH_3_CH_2_-CH_2_-CH_2_-,m; 4.14−4.10 (m, CH_2_OP(O)(OCH_2_CH_2_CH_2_CH_3_)OCH_2_), 3.66−3.58 (m, CH_2_OCH_2_).

^13^C{H} (CD_3_OD), δ (ppm): 70.43 (CH_2_OCH_2_), 70.03 (d, ^3^J(P,C) = 5.8 Hz, POCH_2_CH_2_), 64.57 (d, ^2^J(P,C) = 6.2 Hz, POCH_2_CH_2_); 14.04 (CH_3_CH_2_CH_2_CH_2_-); 18.94 (CH_3_CH_2_CH_2_CH_2_-); 35.03 (CH_3_CH_2_CH_2_CH_2_-).

^31^P{H} NMR (CD_3_OD): δ (ppm):−0.22 (1.98%); −0.17 (96.4%); −0.9 (1.64%).

^31^P NMR (CD_3_OD): δ (ppm): −0.22 m, CH_3_OP(O)(OCH_2_CH_2_CH_2_CH_3_)OCH_2_-. −0.17, septet, ^3^J(P,H) = 10.38 Hz, -CH_2_OP(O)(OCH_2_CH_2_CH_2_CH_3_)OCH_2_-; −0.9 m, −CH_2_OP(O)(OCH_2_CH_2_CH_2_CH_3_)OH;

IR (KBr, cm^−^^1^): 1252 (P=O) (stretching); 1133 (P-OCH_2_-, (CH_2_OCH_2_) (stretching); 2843–2936 (stretching); CH_3_CH_2_CH_2_.

Spectroscopic data for poly(octyloxyethylene phosphate) based on PEG 400:

^1^H NMR (CD_3_OD), δ (ppm): 0.89, CH_3_CH_2_-(t, ^3^J(H,H) = 5.7 Hz); 1.22–1.26, CH_3_(CH_2_)_4_-CH_2_-(m); 1.48–1.53, CH_3_(CH_2_)_4_-(CH_2_)_2_-CH_2_-,m; 3.66–3.58 (m, CH_2_OCH_2_). 4.14–4.10 (m, -CH_2_OP(O)(OCH_2_CH_2_(CH_2_)_2_-(CH_2_)_4_-CH_3_)OCH_2_-).

^13^C{H} (CD_3_OD), δ (ppm): 70.43 (CH_2_OCH_2_), 70.03 (d, ^3^J(P,C) = 5.8 Hz, POCH_2_CH_2_), 64.57 (d, ^2^J(P,C) = 6.2 Hz, POCH_2_CH_2_); 14.04 [CH_3_(CH_2_)_4_(CH_2_)_2_CH_2_-]; 22.09 (CH_3_CH_2_CH_2_-); 25.30 [CH_3_CH_2_(CH_2_)_2_ CH_2_CH_2_CH_2_CH_2_-]; 29.12 [CH_3_CH_2_(CH_2_)_2_ CH_2_CH_2_CH_2_CH_2_-]; 31.89 [CH_3_CH_2_(CH_2_)_2_ CH_2_ (CH_2_)_2_CH_2_-]; 32.79 [CH_3_ (CH_2_)_4_ CH_2_CH_2_CH_2_-)].

^31^P{H} NMR (CD_3_OD): δ (ppm): −0.25 (1.98%); −0.19 (95.9%); −0.7 (1.64%).

^31^P NMR (CD_3_OD): δ (ppm): −0.27, m, CH_3_OP(O)(OCH_2_(CH_2_)_2_(CH_2_)_4_CH_3_)OCH_2_ −0.19, m, -CH_2_OP(O)(OCH_2_(CH_2_)_2_(CH_2_)_4_CH_3_)OCH_2_-; −0.7, m, −CH_2_OP(O)(OCH_2_(CH_2_)_2_(CH_2_)_4_CH_3_)OH; 

IR (KBr, cm^−1^): 1252 (P=O) (stretching); 1133 (P-OCH_2_-, (CH_2_OCH_2_) (stretching); 2843–2936 (stretching); CH_3_(CH_2_)_4_(CH_2_)_2_-.

Spectroscopic data for poly(decyloxyethylene phosphate) based on PEG 400:

^1^H NMR (CD_3_OD), δ (ppm): 0.89, CH_3_CH_2_-(t, ^3^J(H,H) = 5.7 Hz); 1.22–1.26, CH_3_(CH_2_)_8_-CH_2_-(m); 1.48–1.53, CH_3_(CH_2_)_6_-(CH_2_)_2_-CH_2_-,m; 3.66–3.58 (m, CH_2_OCH_2_). 4.14–4.10 (m, -CH_2_OP(O)(OCH_2_(CH_2_)_4_-(CH_2_)_4_-CH_3_)OCH_2_-).

^13^C{H} (CD_3_OD), δ (ppm): 70.43 (CH_2_OCH_2_), 70.03 (d, ^3^J(P,C) = 5.8 Hz, POCH_2_CH_2_), 64.57 (d, ^2^J(P,C) = 6.2 Hz, POCH_2_CH_2_); 14.04 [CH_3_(CH_2_)_6_(CH_2_)_2_CH_2_-]; 22.09 (CH_3_CH_2_CH_2_-); 25.30 [CH_3_CH_2_(CH_2_)_4_ CH_2_CH_2_CH_2_CH_2_-]; 29.12 [CH_3_CH_2_(CH_2_)_4_ CH_2_CH_2_CH_2_CH_2_-]; 30.69 [CH_3_CH_2_(CH_2_)_4_CH_2_ (CH_2_)_2_CH_2_-]; 32.79 [CH_3_ (CH_2_)_4_ (CH_2_)_3_CH_2_-CH_2_-)].

^31^P{H} NMR (CD_3_OD): δ (ppm): −0.26 (1.08%); −0.18 (96.4%); −0.75 (1.54%).

^31^P NMR (CD_3_OD): δ (ppm −0.18, septet, ^3^J(P,H) = 10.45 Hz, −CH_2_OP(O)(OCH_2_(CH_2_)_3_(CH_2_)_5_CH_3_)OCH_2_−.

IR (KBr, cm^−^^1^): 1252 (P=O) (stretching); 1133 (P-OCH_2_-, (CH_2_OCH_2_) (stretching); 2843–2936 (stretching); CH_3_(CH_2_)_5_(CH_2_)_3_-.

Spectroscopic data for poly(dodecyloxyethylene phosphate) based on PEG 400:

^1^H NMR (CD_3_OD), δ (ppm): 0.89, CH_3_CH_2_-(t, ^3^J(H,H) = 5.7 Hz); 1.22–1.26, CH_3_(CH_2_)_10_-CH_2_-(m); 1.48–1.53, CH_3_(CH_2_)_8_-(CH_2_)_2_-CH_2_-,m; 3.66–3.58 (m, CH_2_OCH_2_). 4.14–4.10 (m, -CH_2_OP(O)(OCH_2_(CH_2_)_10_-CH_3_)OCH_2_-).

^13^C{H} (CD_3_OD), δ (ppm): 70.43 (CH_2_OCH_2_), 70.03 (d, ^3^J(P,C) = 5.8 Hz, POCH_2_CH_2_),

64.57 (d, ^2^J(P,C) = 6.2 Hz, POCH_2_CH_2_); 14.04 [CH_3_(CH_2_)_8_(CH_2_)_2_CH_2_-]; 22.09 (CH_3_CH_2_CH_2_-); 25.30 [CH_3_CH_2_(CH_2_)_5_ (CH_2_)_4_CH_2_-]; 29.12 [CH_3_CH_2_(CH_2_)_5_(CH_2_)_3_CH_2_]; 32.79 [CH_3_ (CH_2_)_6_ (CH_2_)_3_CH_2_ -CH_2_-)].

^31^P{H} NMR (CD_3_OD): δ (ppm): = −0.28 (1.28%); −0.21 (92.7%); −0.12 (5.72%).

^31^P NMR (CD_3_OD): δ (ppm): −0.21, septet, ^3^J(P,H) = 10.30 Hz, -CH_2_OP(O)(OCH_2_(CH_2_)_5_(CH_2_)_5_CH_3_)OCH_2_-.

IR (KBr, cm^−1^): 1252 (P=O) (stretching); 1133 (P-OCH_2_-, (CH_2_OCH_2_) (stretching); 2843–2936 (stretching); CH_3_(CH_2_)_7_(CH_2_)_4_-.

### 3.5. Measurement of Polymeric Micelle Size

The synthesized polymer was dissolved in distilled water and exposed to ultrasonic irradiation for 10 min to form micelles. First, to examine the effects of the polymer structure on micelle formation, polymeric micelle size in 5 mg/mL (0.5 wt%) polymer solutions was prepared by the above method was measured by DLS at 25 °C. Next, to study the stability of micelles after the change of concentration, 10 mg/mL polymer solutions were measured by DLS at 25 °C. After that, the solutions were diluted by 5.0, 2.5, 1.0, and 0.1 mg/mL and measured with DLS under the same conditions. Finally, to investigate the stability of micelles from the temperature, 5 mg/mL of polymer solution at 25, 30, 35, and 40 °C was prepared and measured with DLS at the same temperature.

### 3.6. Solubilizing Test

Polymer 50 mg and Sudan III 10 mg were added to a round-bottomed flask and dissolved in 10 mL of distilled water. Then, the solution was exposed to ultrasonic irradiation for 10 min to form micelle and to incorporate hydrophobic pigment. Finally, it was filtered through a 0.45 μ syringe-driven filter to remove free pigment and aggregation substances. The absorption spectra of the, thus, obtained solution were measured with a UV-visible spectrophotometer. After that, 5 mL of sample was taken from the solution and freeze-dried to remove water. Then, 5 mL of chloroform was added to dissolve residual materials. The absorbance of the prepared solution was measured with the UV-visible spectrophotometer at 500 nm. The solubilizing capacity was calculated by dividing the amount of pigment estimated from the absorbance by the amount of polymer used.

## 4. Conclusions

Using a novel strategy, amphiphilic polyphosphoesters, based on poly(oxyethylene H-phosphonate)s (POEHP) with different lengths of poly(ethylene glycol) segments and aliphatic alcohols with different alkyl chain lengths, were synthesized using polycondensation reactions. Poly(alkyloxyethylene phosphate)s with an alkyl side chain length greater than or equal to the octyl group were self-assembled into the micellar structure in an aqueous solution. Poly(alkyloxyethylene phosphate)s with long PEG chain lengths had a low coagulation powder and dissolved in water. By heating the polymer solution of these poly(alkyoxyethylene phosphate)s, they gradually assembled. Polymeric micelles were relatively monodispersed and small enough to deliver the drug into tumor tissue via EPR effects. They are capable of encapsulating hydrophobic guest molecules, such as Sudan III. Polyphosphoesters with longer hydrophobic side chains form smaller and more stable micelles and dissolve more pigment. The results obtained indicate that these structurally flexible amphiphilic polyphosphoesters have the potential as carriers of hydrophobic drugs.

## Figures and Tables

**Table 1:** Characteristics of poly(oxyethylene H-phosphonate)s (POEHP).

Entry	Yield (%)	M*_n_* (×10^3^) ^1^	M*_w_* (×10^3^) ^1^	M*_w_*/M*_n_* (PDI) ^1^	DP
P_200_-H	84.3	3.6	6.6	1.83	14.8
P_300_-H	86.8	6.4	8.5	1.32	18.6
P_400_-H	84.2	11.5	15.5	1.34	25.8
P_600_-H	80.1	12.4	17.4	1.40	19.2

^1^ Determined by SEC.

**Table 2:** Reaction time of the alkylation of POEHP.

P*_a_*-*b*	Reagent	Reaction Time (h)
P_400_-OCH_3_	methanol	3
P_400_-OC_4_H_9_	1-butanol	5
P_400_-OC_8_H_17_	1-octanol	15
P_400_-OC_10_H_21_	1-decanol	20
P_400_-OC_12_H_25_	1-dodecanol	24

**Table 3:** Dependence of micelle diameter of POEAP from PEG’s length.

Entry P*_a_-b*	Degree of Alkylation ^1^ (%)	Micelle Diameter ^2^ (nm)
P_200_-OC_12_H_25_	95	102.7 ± 25.0
P_300_-OC_12_H_25_	93	93.6 ± 17.5
P_400_-OC_12_H_25_	93	93.8 ± 18.2
P_600_-OC_12_H_25_	90	2.5 ± 0.4

^1^ Determined by 3^1^P{H}NMR. ^2^ Determined by DLS (number).

**Table 4:** Dependence of micelle diameter of POEAP from alkyl chain length.

Entry P*_a_-b*	Degree of Alkylation ^1^ (%)	Micelle Diameter ^2^ (nm)
P_300_-OC_8_H_17_	92	145.3 ± 34.0
P_300_-OC_10_H_21_	92	123.7 ± 28.4
P_300_-OC_12_H_25_	93	93.6 ± 17.5

^1^ Determined by ^31^P{H}NMR. ^2^ Determined by DLS (number).

**Table 5:** Dependence of micelle diameter from the alkyl chain length and polymer concentration.

Entry P*_a_-b*	Diameter ^1^ (nm)				
	1.0 wt%	0.5 wt%	0.25 wt%	0.1 wt%	0.01 wt%
P_400_-OC_8_H_17_	340.1 ± 81.8	183.2 ± 44.2	158.0 ± 41.9	165.7 ± 42.6	106.0 ± 23.9
P_400_-OC_10_H_21_	173.2 ± 30.9	170.2 ± 29.8	165.6 ± 23.3	165.2 ± 23.0	170.4 ± 29.1
P_400_-OC_12_H_25_	101.9 ± 26.3	93.8 ± 18.2	92.2 ± 16.4	80.3 ± 14.7	94.6 ± 18.3

^1^ Determined by DLS (number) at 30 °C.

**Table 6:** Dependence of micelle diameter on the temperature and PEG’s chain length.

Entry P*_a_-b*	Diameter ^1^ (nm)			
	25 °C	30 °C	35 °C	40 °C
P_200_-OC_12_H_25_	118.3 ± 23.9	104.7 ± 23.1	100.7 ± 21.6	95.0 ± 18.5
P_300_-OC_12_H_25_	120.0 ± 30.4	124.4 ± 29.1	123.5 ± 28.5	122.8 ± 26.8
P_400_-OC_12_H_25_	1.3 ± 0.1	116.4 ± 22.0	73.6 ± 16.7	64.9 ± 13.7
P_600_-OC_12_H_25_	2.5 ± 0.4	1.4 ± 0.2	29.1 ± 7.5	18.8 ± 5.4

^1^ Determined by DLS (number) at 25 °C.

**Table 7:** Dependence of solubilizing ability of poly(alkyloxyethylene phosphate) based on PEG 300 from alkyl chain length. The value of solubilizing capacity was calculated for the polymers for which the size of the micelle could be measured.

Entry P*_a_-b*	Abs. (500 nm)	Solubilizing Capacity (%)
P_300_-OC_12_H_25_	1.332	0.21
P_300_-OC_10_H_21_	0.920	0.18
P_300_-OC_8_H_17_	0.637	0.11
P_300_-OC_4_H_9_	0.091	-
P_300_-OCH_3_	0.015	-
Only water	0.004	-

## Data Availability

The data presented in this study are available in the Supplementary Materials.

## Supplementary Materials

The following supporting information can be downloaded at: https://www.mdpi.com/article/10.3390/molecules27186006/s1. Figure S1: ^1^H NMR spectrum of poly(oxyethylene H-phosphonate), based on PEG 400. Figure S2: ^13^C{H} NMR spectrum of poly(oxyethylene H-phosphonate), based on PEG 400. Figure S3: ^31^P{H}NMR spectra of poly(oxyethylene H-phosphonate), based on PEG 400. Figure S4: ^31^P NMR spectra of poly(oxyethylene H-phosphonate), based on PEG 400. Figure S5: ^31^P NMR spectra of poly(chloroxyethylene phosphate), based on PEG 400, signal for phosphorous atom in the repeating units. Figure S6: ^31^P{H} NMR spectra of poly(methyloxyethylene phosphate, based on PEG 400. Figure S7: ^31^P NMR spectrum of poly(methyloxyethylene phosphate), based on PEG 400.
