# Supplementary material for: Thermoresponsive Polyphosphoester via Polycondensation Reactions: Synthesis, Characterization, and Self-Assembly
Source: Molecules. 2022 Sep 15;27(18):6006. doi: 10.3390/molecules27186006 (PMC9505623; doi:10.3390/molecules27186006)
Supplement: Supplementary file 1 [file molecules-27-06006-s001.zip › molecules-1870048-supplementary.pdf]

Thermoresponsive Polyphosphoester via Polycondensation Reactions: Synthesis, Characterization, and Self-Assembly

Yoshihiro Yamakita[1], Issei Tekauchi[1], Kimiko Makino[1], Hiroshi Terada[1], Akihiko Kikuchi[1], Kolio Troev[1,2]

Supplementary Data:

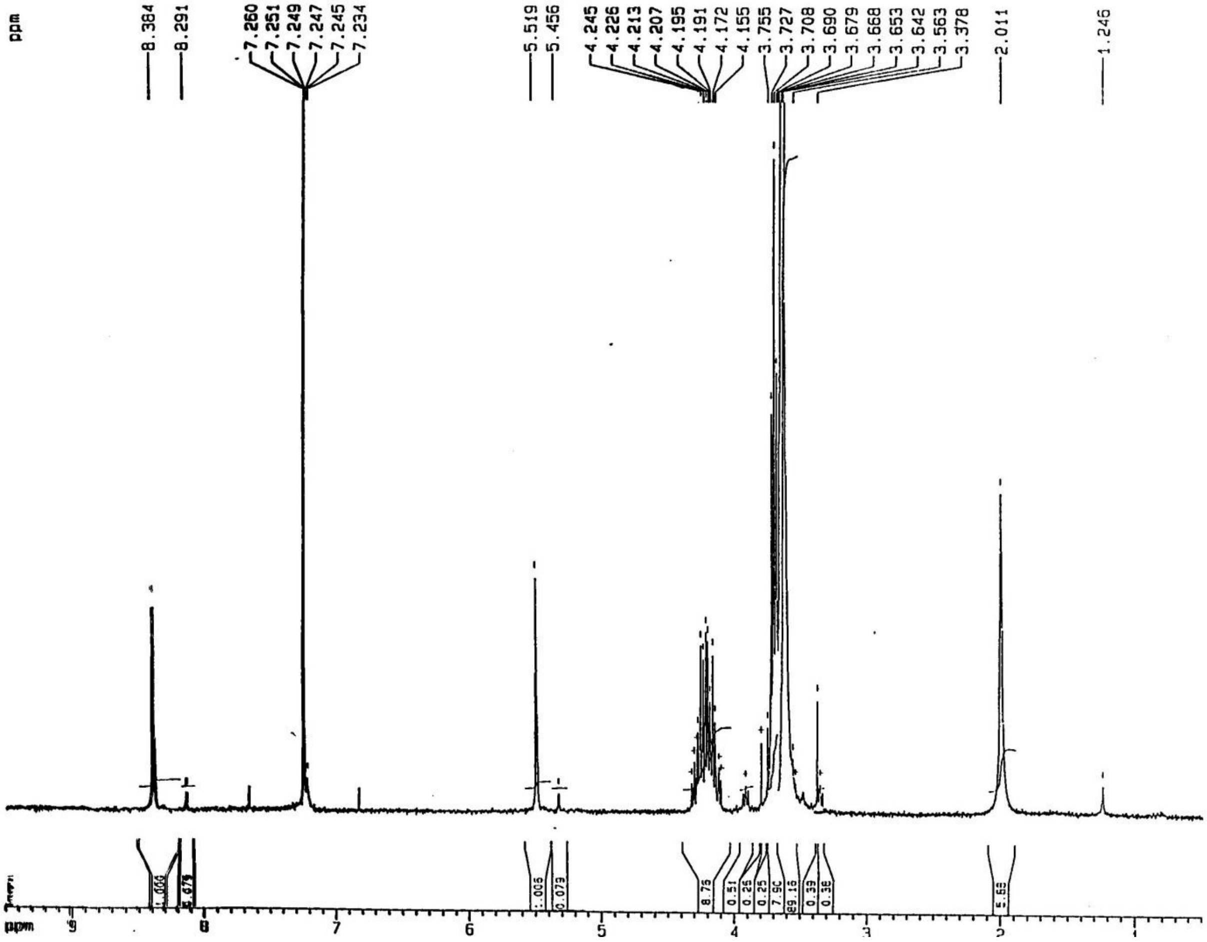

Figure S1. 1H NMR spectrum of poly(oxyethylene H-phosphonate), based on PEG 400.

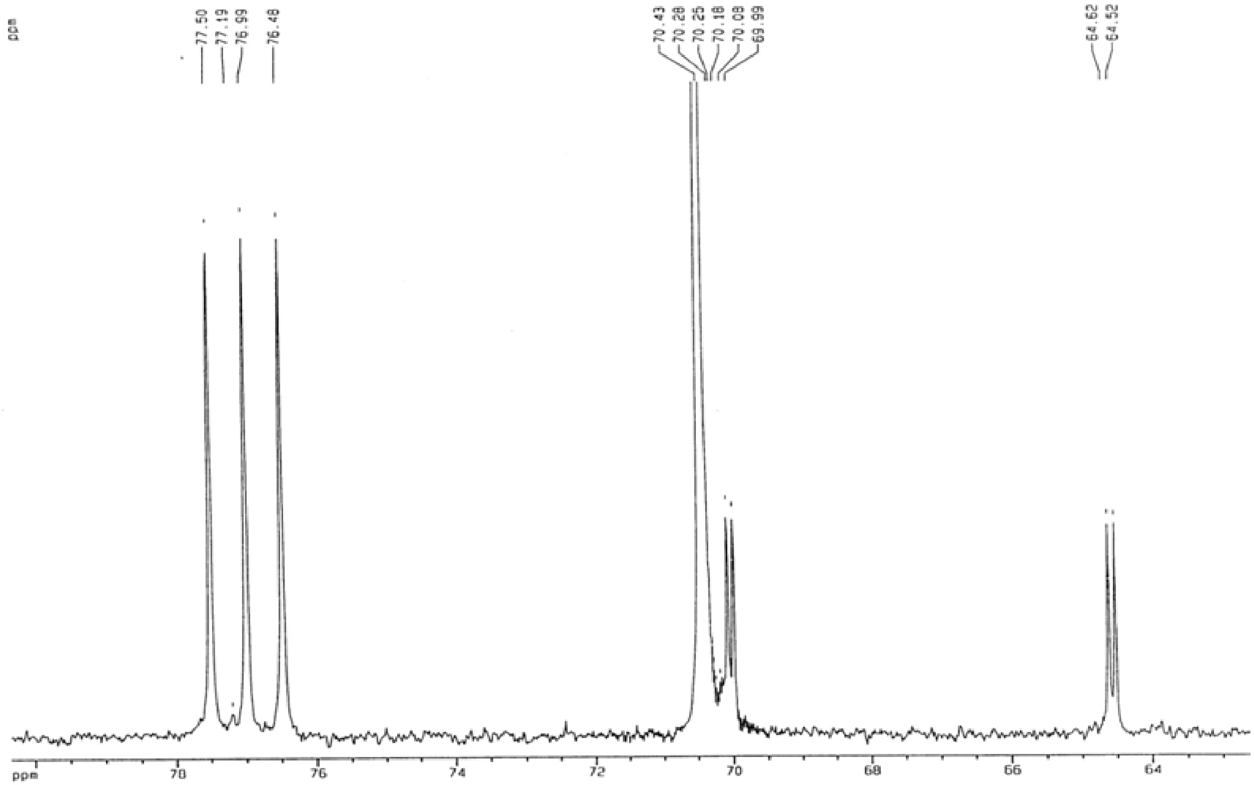

Figure S2. $^{13}C\{H\}$ NMR spectrum of poly(oxyethylene H-phosphonate), based on PEG 400.

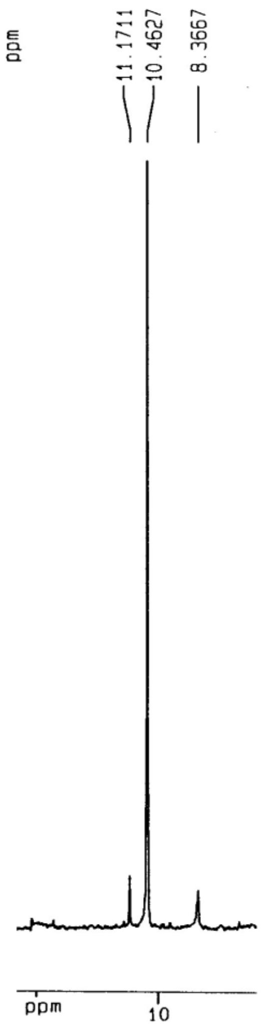

Figure S3. $^{31}P\{H\}$NMR spectra of poly(oxyethylene H-phosphonate), based on PEG 400.

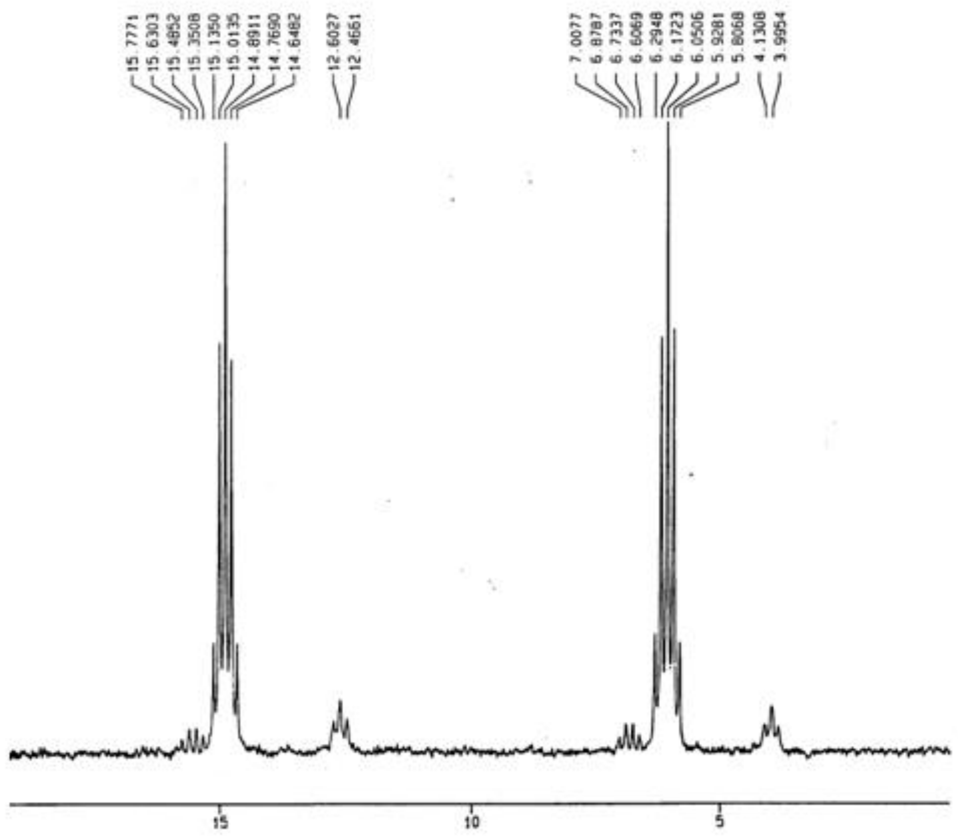

Figure S4. ^{31}P NMR spectra of poly(oxyethylene H-phosphonate), based on PEG 400.

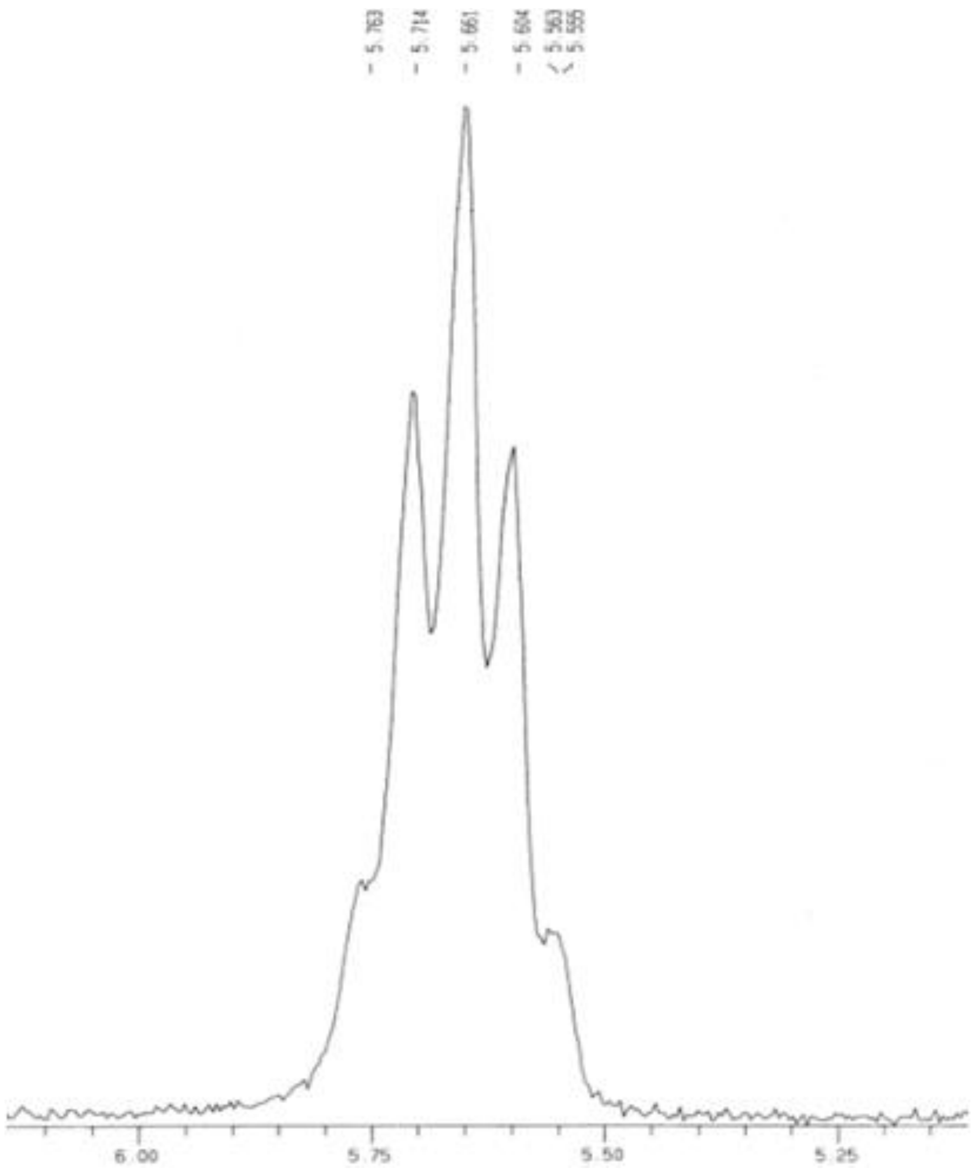

Figure S5. ^{31}P NMR spectra of poly(chloroxyethylene phosphate), based on PEG 400.Signal for phosphorous atom in the repeating units.

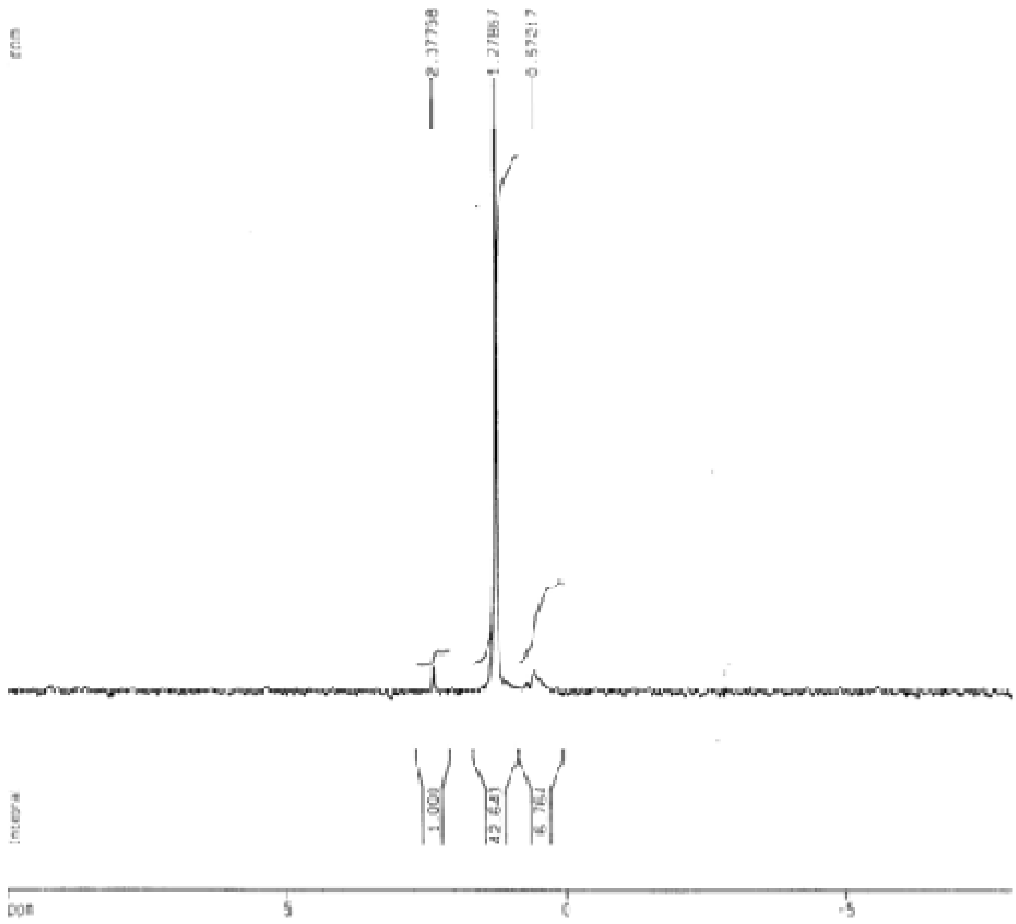

Figure S6. ^{31}P{H} NMR spectra of poly(methyloxyethylene phosphate, based on PEG 400.

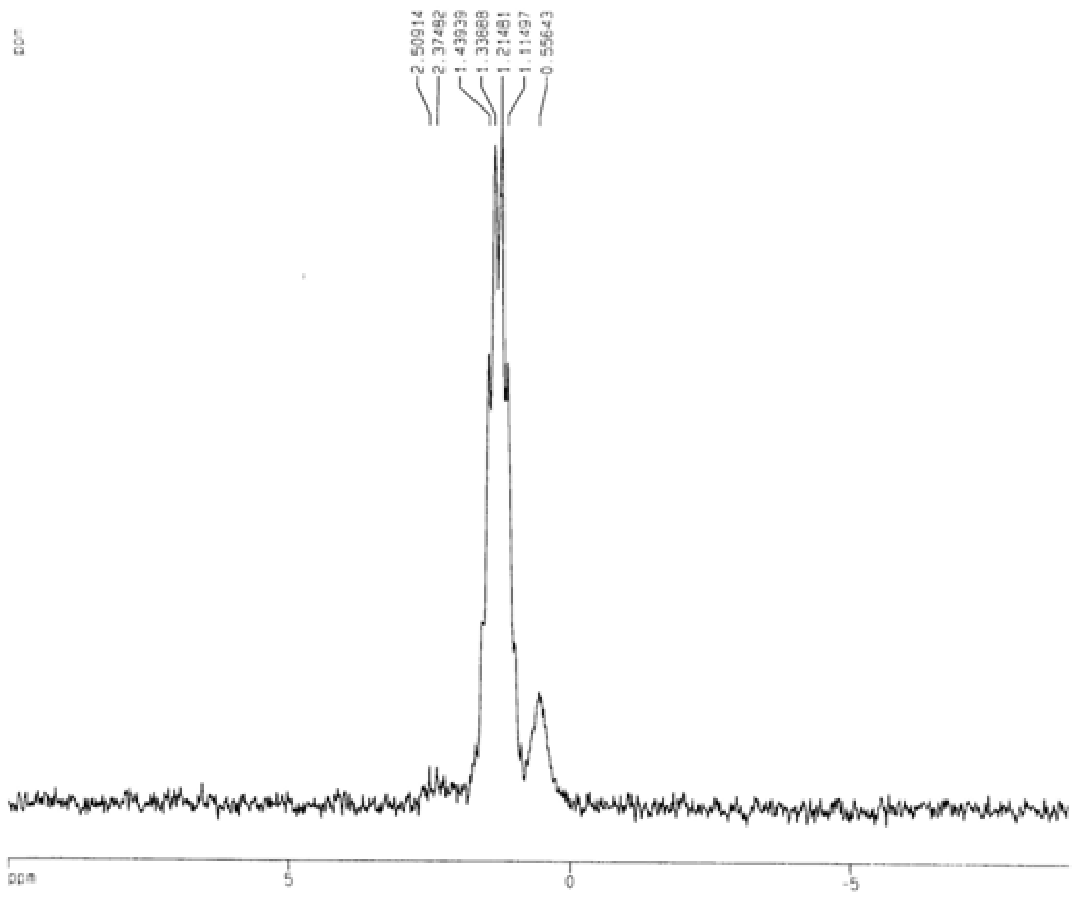

Figure S7. ^{31}P NMR spectrum of poly(methyloxyethylene phosphate), based on PEG 400.
